# Correction: An indenocarbazole-based host material for solution processable green phosphorescent organic light emitting diodes

**DOI:** 10.1039/d1ra90147h

**Published:** 2021-09-15

**Authors:** Eun Young Park, Da Hwan Lee, Thi Na Le, Chol-Min Shin, Jihoon Lee, Min Chul Suh

**Affiliations:** Department of Information Display, Kyung Hee University Dongdaemun-gu Seoul 02447 Republic of Korea mcsuh@khu.ac.kr; Department of Polymer Science and Engineering, Department of IT·Energy Convergence (BK21 FOUR), Korea National University of Transportation Chungju 27469 Republic of Korea jihoonli@ut.ac.kr

## Abstract

Correction for ‘An indenocarbazole-based host material for solution processable green phosphorescent organic light emitting diodes’ by Eun Young Park *et al.*, *RSC Adv.*, 2021, **11**, 29115–29123. DOI: 10.1039/D1RA04855D.

The authors regret that an incorrect version of Fig. 1 was included in the original article. The correct version of Fig. 1 is presented below.

**Fig. 1:**
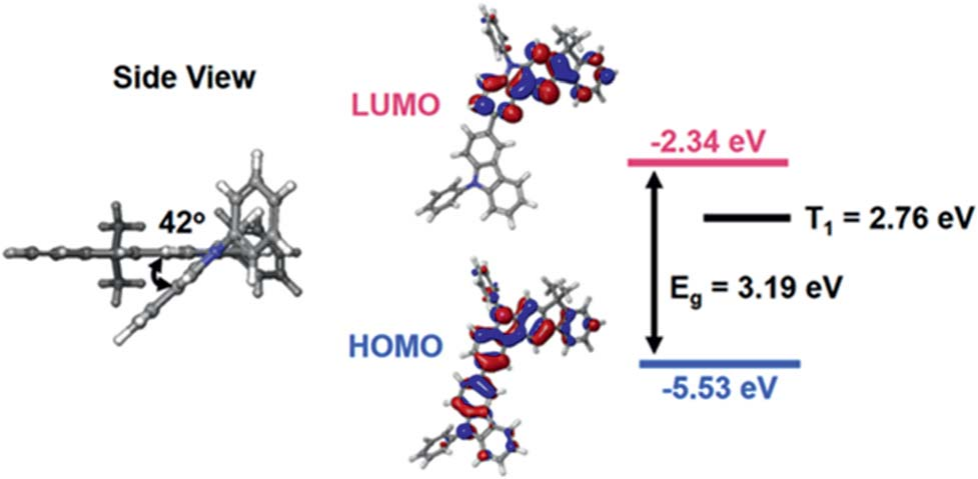
HOMO, LUMO distributions and energy level of PCIC predicted through DFT and TD-DFT calculations.

The Royal Society of Chemistry apologises for these errors and any consequent inconvenience to authors and readers.

## Supplementary Material

